# Two Dimensional Heterostructures for Optoelectronics: Current Status and Future Perspective

**DOI:** 10.3390/molecules28052275

**Published:** 2023-02-28

**Authors:** Zaheer Ud Din Babar, Ali Raza, Antonio Cassinese, Vincenzo Iannotti

**Affiliations:** 1 Scuola Superiore Meridionale (SSM), University of Naples Federico II, Largo S. Marcellino 10, 80138 Naples, Italy; 2 Department of Physics “Ettore Pancini”, University of Naples Federico II, Piazzale Tecchio 80, 80125 Naples, Italy; 3 CNR–SPIN (Institute for Superconductors, Oxides and Other Innovative Materials and Devices), Piazzale V. Tecchio 80, 80125 Naples, Italy

**Keywords:** optoelectronics, mechanical transfer, chemical synthesis, 2D heterostructures, photodetection, photovoltaics

## Abstract

Researchers have found various families of two-dimensional (2D) materials and associated heterostructures through detailed theoretical work and experimental efforts. Such primitive studies provide a framework to investigate novel physical/chemical characteristics and technological aspects from micro to nano and pico scale. Two-dimensional van der Waals (vdW) materials and their heterostructures can be obtained to enable high-frequency broadband through a sophisticated combination of stacking order, orientation, and interlayer interactions. These heterostructures have been the focus of much recent research due to their potential applications in optoelectronics. Growing the layers of one kind of 2D material over the other, controlling absorption spectra via external bias, and external doping proposes an additional degree of freedom to modulate the properties of such materials. This mini review focuses on current state-of-the-art material design, manufacturing techniques, and strategies to design novel heterostructures. In addition to a discussion of fabrication techniques, it includes a comprehensive analysis of the electrical and optical properties of vdW heterostructures (vdWHs), particularly emphasizing the energy-band alignment. In the following sections, we discuss specific optoelectronic devices, such as light-emitting diodes (LEDs), photovoltaics, acoustic cavities, and biomedical photodetectors. Furthermore, this also includes a discussion of four different 2D-based photodetector configurations according to their stacking order. Moreover, we discuss the challenges that remain to be addressed in order to realize the full potential of these materials for optoelectronics applications. Finally, as future perspectives, we present some key directions and express our subjective assessment of upcoming trends in the field.

## 1. Introduction

Since its mechanical exfoliation in 2004, graphene has drawn much consideration and earned a colossal reputation as the “*wonder material*”. Various features, such as ultra-high-carrier mobility [1], a large active surface [2], superior in-plane thermal conductivity, and comparatively better mechanical properties (a Young’s modulus of 1 TPa and intrinsic strength of 130 GPa) [3], have made graphene a viable material for numerous applications. For instance, this includes applications such as ultrafast high-frequency photodetectors and transparent electrodes [4,5]. However, the reduced energy bandgap in graphene limits research as graphene-based transistors often have a low on/off current ratio [6]. In the meantime, several other 2D materials, e.g., WTe_2_ [7,8], Pb_1−*x*_Sn*_x_*Te, Bi_2_Te_3_ [9,10], black phosphorous (BP), MoS_2_ [11], WS_2_ [12], WSe_2_ [13], and boron nitride (BN) [14], possess varying bandgaps and have been studied extensively. In addition to graphene’s zero-band gap, 2D materials have different bandgaps that can be further tuned by varying the thickness of layers. This has drawn significant interest in the scientific community and refocused the attention toward the fabrication of novel structures. The thickness-dependent bandgaps of such 2D materials lead to their applications in several devices, namely, photodetectors [15,16,17], field-effect transistors [18,19], and flexible electronics [20,21,22], to name a few. Furthermore, such 2D material facilitates atomic-scale integration, enabling advanced heterostructure devices with new physics and better technological aspects [23,24]. Generally, 2D material-based vdWHs can be produced via chemical-vapor deposition (CVD) or mechanical-transfer growth strategies [25,26]. The typical semiconductor-based heterostructures need comparable lattice structures among corresponding component semiconductors. In comparison, vdWHs have weak interaction between layers, so they have less stringent lattice-mismatching requirements [27,28].

It is imperative to develop and implement non-destructive methods to probe and explore the physicochemical and structural properties of 2D layered materials. Considering their small size, 2D nanostructured materials are fascinating in terms of their vibrational and molecular structure. Since the electrons have a short de Broglie wavelength, it allows for better spatial resolution. Similarly, electron microscopy limits the resolution of a light microscope to about 1 μm. In that respect, SEM and TEM are two popular microscopy techniques [29]. These approaches can offer detailed information on the crystalline nature and corresponding relationships between layer thickness, layer spacing, and element arrangement [30,31,32,33]. For example, selected-area electron diffraction (SAED) can discriminate a monolayer or a multilayer of graphene depending on the diffraction-intensity ratio from 110 to 100 [34]. Furthermore, the unique diffraction pattern can be identified when the interlayer spacing changes within a monolayer or multilayer of a 2D material.

On the other hand, Raman spectroscopy involves the inelastic scattering of monochromatic light, which is mainly categorized into Stokes and anti-Stokes scattering (emitted energy is larger than incoming energy). It involves the scattering of incoming light at different wavelengths than the initial wavelength when the sample is irradiated [35]. By comparing the incoming and outgoing light, vibrational modes can be determined, which are then used to create a fingerprint of each material. Photoluminescence (PL) measurements are the most commonly employed tool to characterize the electronic structure [29]. The radiative recombination of the electron–hole pair may be preceded by a non-radiative relaxation mechanism, leading to phonon emission. Comparing the non-radiative and radiative processes is vital to determine the characteristics of most 2D nanostructured materials. Multiple microscopy methods are applied to examine the optical characteristics of 2D materials, which are greatly influenced by their physical structure. It is commonly used to study morphology and topography using AFM, SEM, ultrasonic force microscopy (UFM), and TEM. In many cases, AFM can provide a layer-thickness measurement with preciseness as low as 5% [31,36]. In contrast, anomalies occur when the tip interacts differently with the substrate and the stacked layers [37]. However, the distance between the tip and the surface from any hysteretic parts is crucial to controlling these discrepancies and obtaining the ideal height profile of a single layer [37]. To determine the thickness of a single layer precisely; it is preferable to compare the heights of the first two layers instead of recording the difference in height between the substrate and the first layer.

When it comes to evaluating mechanical properties, such as mechanical interaction with substrates, ultrasonic force microscopy (UFM) is an effective method of assessing stiffness at the nanoscale [38,39]. Precisely, the sample is vibrated using a low vibrational amplitude (0.5–2 nm) and high vibrational frequencies (0.10–10 MHz) that are higher than the cantilever resonance frequencies of the AFM. It is possible to identify subsurface structures, including cavities, subsurface interfaces, and sample-substrate interfaces, by analyzing the material’s stiffness. It is possible to monitor ultrasonic vibration at the tip–sample junction on a separate channel and quantify it simultaneously with AFM using nonlinear rectification [29].

The present review focuses on the existing state-of-the-art concerning the material design, fabrication routes, and strategies to design novel heterostructures. Distinct fabrication approaches, e.g., mechanical transfer and chemical synthesis, are discussed extensively. In the meantime, critical factors and controlling parameters in each fabrication approach are also highlighted. Electric and optical features of vdWHs are thoroughly reviewed, with a particular emphasis on energy-band alignments. Later on, several optoelectronic applications of 2D vdWHs, such as photodetectors in biomedical fields, LEDs, photovoltaics, and acoustic cavities, are also reviewed. Notably, we explain four different configurations of 2D material-based photodetectors according to the stacking fashion of the layers. As future perspectives, we present some key directions and express our subjective assessment of upcoming trends.

## 2. Current Status

**Figure 1:**
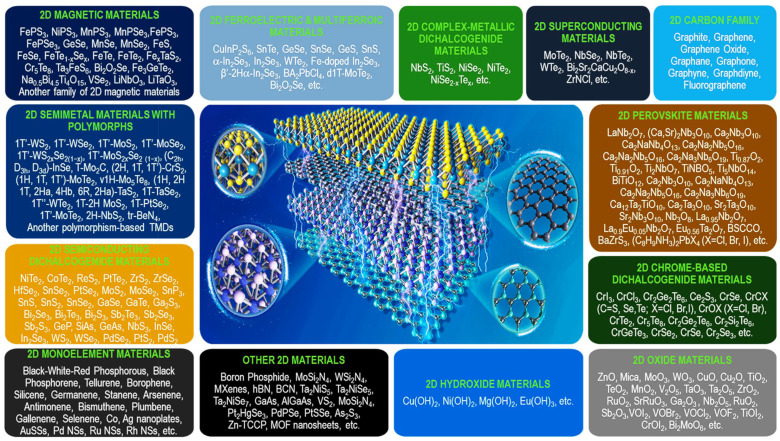
Summary of the current status of 2D materials presented to date. Adapted with permission from Ref. [40]. Copyright 2022, American Chemical Society.

Two-dimensional stacked crystals, also called 2D heterostructures, have become increasingly popular over the past few decades. The diversity of their chemistry and physics has fascinated researchers and led to developing new perspectives. Similar efforts have facilitated the growth of 2D heterostructures with interesting thermal, optical, and electrical properties. Two-dimensional materials with weak vdW interactions among layers can be exfoliated into isolated atomic layers. Such isolated layers can further be rearranged into horizontally and vertically stacked heterostructures. In addition to conventional indirect growth of 2D crystals, current developments in vapor-phase deposition provide more opportunities for direct growth to realize such heterostructures (e.g., in-plane and vertically stacked). Therefore, 2D heterostructures produced by stacking semiconductors engender further opportunities in the semiconductor industry. This leads to applications such as highly efficient LEDs, photodetectors, neuromorphic devices, solar cells, lasers, and many others. Two-dimensional heterostructures are typically fabricated by combining two or more semiconductors, providing distinct electronic-band structures at the interface. Generally, a class of materials with identical elements, such as carbon materials (g-C_3_N_4_ and graphene), and/or transition-metal dichalcogenides (TMDs) such as MoS_2_ and WS_2_, etc., or similar attributes of individual elements, can be grouped into a particular classification. As guidance, we considered a clear taxonomy based on the latest advances that could prove helpful for the scientific society to foresee and delve into the following research fields (Figure 1).

**Figure 2:**
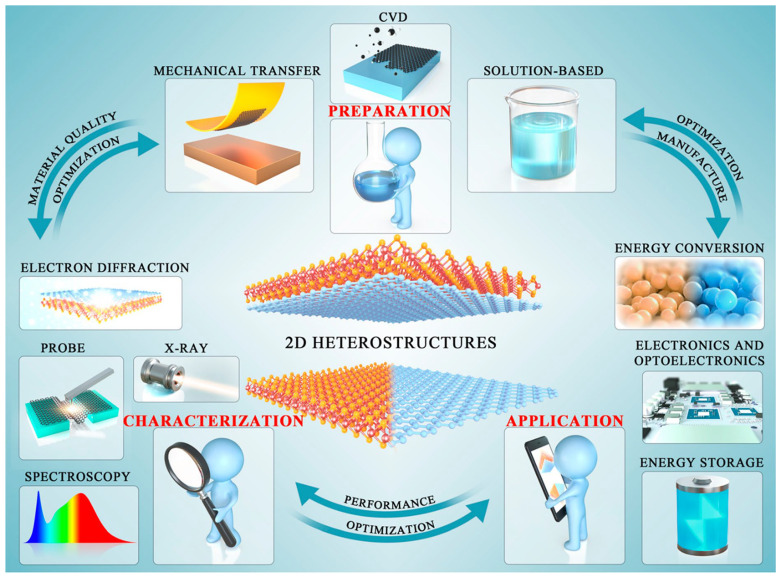
Characteristic approaches executed for research on 2D heterostructures. Adapted with permission from Ref. [40]. Copyright 2022, American Chemical Society.

Recently, the discovery of numerous 2D materials has radically changed the dynamics of semiconductor junctions. We expect this advancement to be an appropriate approach to representing several 2D materials discovered so far. The 2D-material family contains the majority of members, e.g., 2D carbon families [24,41]; magnetic materials, particularly their derivatives [42,43]; superconductors [44,45]; perovskites [46]; semiconducting dichalcogenides [47,48]; complex metallic dichalcogenides [49,50]; chrome-based dichalcogenides [51,52,53]; semimetals with polymorphs [54,55], in particular, polymorphism-based TMDs [56,57,58]; and mono-elemental materials, also known as “homonuclear materials” [59,60]. Moreover, it includes 2D ferroelectric and multiferroic materials [61,62,63], oxides [64,65], hydroxides [66,67], and other 2D materials (Figure 1) [68,69]. In future electronics and optoelectronics applications, developing these 2D materials will make it easier to build novel heterostructures with improved efficiency. Generally, different 2D materials are prone to certain limitations in particular devices. However, it has been observed that synergistic effects become visible when combined with other 2D materials or reassembled in stacked layers, resulting in a complete, robust material with superior performance. Aligned transfer, mechanical exfoliation, liquid-phase exfoliation, CVD, layer-by-layer (LbL), and electrostatic self-assembly are some of several approaches used to fabricate 2D heterostructures with unalike junctions/interfaces. In addition, sophisticated characterization techniques have allowed the designing of new 2D heterostructures with distinct heterointerfaces/junctions (Figure 2). Primarily, the fabrication of heterostructures from materials with different dimensions (2D/3D, 2D/2D, and 2D/1D) ensures substantial potential to improve the functions of futuristic optoelectronics. Still, scalable production of low-dimensional materials is critically important to reach the theoretical extent of performance. The requirement of advanced and controllable scalable transfer approaches to grow distinct integrated vdWHs with high quality impedes their true potential. Therefore, it is necessary to design a reliable procedure to transfer low-dimensional materials on a broader scale and ensure exceptional performance.

## 3. Electronic and Optical Features in vdW Heterostructures

### 3.1. Electronic Properties

**Figure 3:**
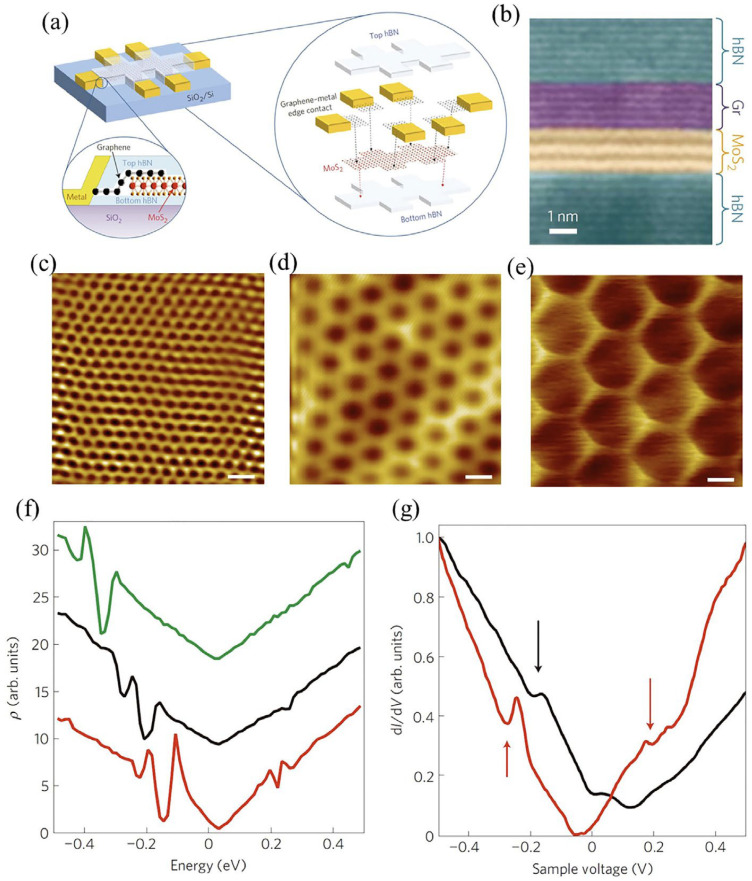
(**a**) Schematic depiction of MoS_2_-based multi-terminal device encapsulated with *h*-BN. (**b**) False-color cross-sectional STEM images of the fabricated device. Adapted with permission from Ref. [77]. Copyright 2015, Nature Publishing Group. STM topography images with moiré patterns at (**c**) 2.4 nm, (**d**) 6.0 nm, and (**e**) 11.5 nm. The scale bar is 5 nm. (**f**) A theoretical low density of state behavior at three distinct rotation angles between graphene and *h*-BN, where green, blue, and red represent 6.3 nm, 10.0 nm, and 12.5 nm, respectively. (**g**) Experimental dI/dV behavior at distinct moiré wavelengths of 13.4 nm (red) and 9.0 nm (black) and the arrows indicate dips in the dI/dV curves. Adapted with permission from Ref. [79]. Copyright 2012, Nature Publishing Group.

The weak vdW interaction among two types of stacked 2D materials significantly changes the electrical properties of adjacent layers. It has been shown that ultra-flat and impurities-free (i.e., chemically pure) *h*-BN can substantially minimize the undesirable substrate effects on graphene [70,71]. Compared with conventional SiO_2_ substrates, graphene on the *h*-BN substrate has a field-effect mobility of about one-fold-larger in magnitude (∼1.4 × 10^5^ cm^2^ V^−1^ s^−1^). In addition, the efficiency can be enhanced even further by encapsulating the graphene, thereby protecting it from surface contamination that could adversely affect its performance [72]. Such engineering approaches and interface variations are prevalent and have proven successful for 2D materials [71,73], especially for air-sensitive BP [74], NbSe_2_ [75], and CrI_3_ [76]. Cui et al. created a MoS_2_ transistor primarily based on vdWH in which graphene electrodes were employed as a source/drain while MoS_2_ layers were contained within *h*-BN, as visualized in Figure 3a,b. The proposed heterostructure attained a superior Hall mobility of ~34,000 cm^2^ V^−1^ s^−1^ at low temperatures [77].

Furthermore, *h*-BN is an optically thin dielectric material capable of withstanding significant electric-field strengths (≥0.5 V per layer) [23]. The main advantage of *h*-BN was to achieve a sophisticated interface. The graphene/*h*-BN heterostructures display unique moiré patterns due to mismatched lattices and misaligned orientations [78]. Lattice constants of *h*-BN and graphene exhibit a discrepancy of 1.8%, sufficient to observe the surface construction. The rotation-dependent moiré pattern can be observed in graphene engineered on *h*-BN according to theoretical analysis and experimental demonstration (Figure 3c–e). Such rotation-dependent moiré patterns function as periodic potentials and trigger the evolution of distinct Dirac points (Figure 3f,g) [79]. A commensurate–incommensurate transformation of graphene/*h*-BN heterostructures was discovered after the substantial alignment of crystallographic orientations of two crystals [80]. It is crucial to note that identical lattice constants separate moiré patterns in regions with relaxed graphene lattices. This effect is diminished whenever elastic energy becomes enormous to be recompensated via vdW in an incommensurate condition. Contrarily, graphene layers twisted at certain angles demonstrate unique physics [81]. Cao et al. reported that double-layer graphene twisted at an angle of 1.1° shows flat bands near zero Fermi energy, thus describing the evolution of insulating states [82]. As a result of electrostatic doping, magic-angle double-layer graphene also exhibits unusual superconductivity with a critical temperature as high as 1.7 K. Analogous to graphene and *h*-BN, precise twisting of one layer over the other, such as the stacked layers of monolayer TMDs (e.g., the layers of MoS_2_/WSe_2_) [83], and several other heterostructures (e.g., MoS_2_/WSe_2_ [83,84], WSe_2_/WS_2_ [85], and MoSe_2_/WS_2_ [86], express intriguing electrical, optoelectronic, and magnetic properties. It shows a considerable promise towards re-engineering the surface in TMD heterostructures.

### 3.2. Optoelectronic Properties

**Figure 4:**
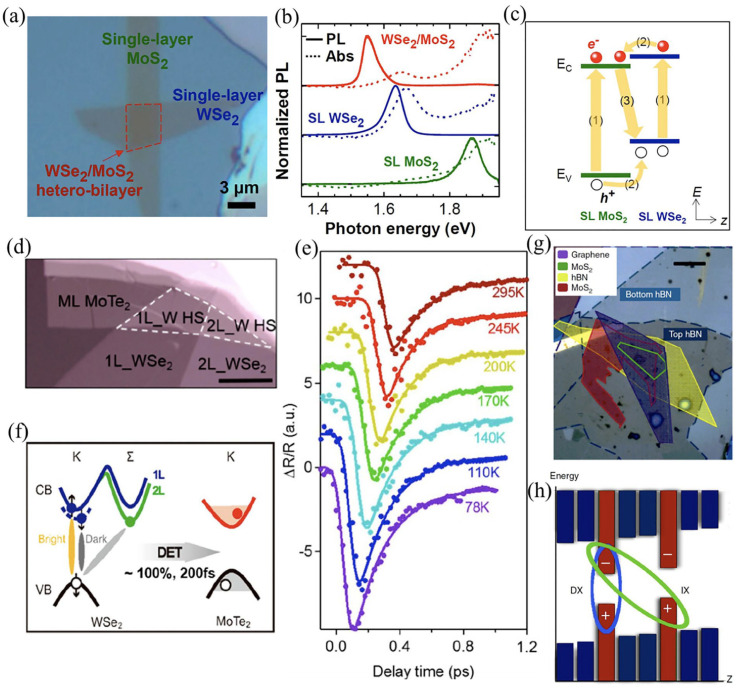
(**a**) Optical microscope images for the hetero-bilayer (WSe_2_/MoS_2_) over the Si/SiO_2_ substrate. (**b**) Normalized PL. (**c**) Energy-band diagram of photoexcited hetero-bilayer WSe_2_/MoS_2_. Adapted with permission from Ref. [91]. Copyright 2014, National Academy of Sciences of the United States of America. (**d**) Characteristic WSe_2_/MoTe_2_ samples with monolayer MoTe_2_ and bilayer WSe_2_, monolayer WSe_2,_ and with their heterostructures for monolayer MoTe_2_. (**e**) Transient reflectance (TR) spectra of WSe_2_ and exciton bleach-recovery kinetics in WSe_2_/MoTe_2_ heterostructures at several temperatures. (**f**) Displays the near-unity transfer of WSe_2_ excitons (dark and bright) into free electrons and holes in the MoTe_2_ monolayer. Adapted with permission from Ref. [95]. Copyright 2019, American Chemical Society. (**g**) Microscopic images of MoS_2_/*h*-BN heterostructure. (**h**) Energy-band mapping of MoS_2_/*h*-BN heterostructure (where IX = indirect exciton; DX = direct exciton). Adapted with permission from Ref. [94]. Copyright 2015, Nature Publishing Group.

In recent decades, extensive research efforts have been devoted to layer-dependent band structures and many other characteristics of several 2D materials [23,87]. Furthermore, vdW stacking can further revamp the optoelectronic features of 2D materials [88,89]. The research on vdW interactions among the layers and dynamic excitons in 2D materials is made possible by rapidly evolving routes such as PL, ultrafast optical, and Raman spectroscopy. Novel modes of lattice vibration could be developed via interfacial interactions at an interface in 2D semimetals and semiconductors-based vdWHs. Li et al. used ultralow-frequency Raman spectroscopy to identify numerous novel characteristics of layer-breathing modes in MoS_2_/graphene-based vdWH [90]. Contrary to high-frequency modes (E_12g_ and A_1g_), these modes are governed via flake thickness and susceptible to interfacial coupling. Moreover, the interfacial force constant for layer-breathing was reported to be α_0_^⊥^ (I) = 60 × 10^18^ Nm^−3^, as determined through the linear chain model. Fang et al. employed a micromachining transfer approach to create a vdW hetero-bilayer heterostructure based on monolayer WSe_2_ and MoS_2_ (Figure 4a) and studied the interlayer carrier coupling using PL and absorbance spectra [91]. The PL spectra shown in Figure 4b correspond to a Stokes-like shift of ~100 meV. This shifting follows the type-II band alignment, which holds spatially indirect emission and absorption (Figure 4c). Upon optical or electrical stimulation, abundant emissive and non-emissive dark excitons are present in 2D vdW optoelectronic structures. The ultra-fast interlayer energy transfer (approx. 200 femtoseconds) studied by Wu et al. in WSe_2_/MoTe_2_ vdWHs exhibited a near-unity PL output. This shows that, in addition to emissive excitons, non-emissive excitons such as spin-forbidden dark excitons and momentum-forbidden indirect excitons also exhibit similar behavior. Furthermore, in vdW layers, the transmission of both an electron and a hole denotes a dominating Dexter-type energy transfer (Figure 4d–f). The primary illustration for ultrafast charge transfer was described by Hong et al. in MoS_2_/WS_2_-based vdWHs. They discovered a rapid (in under 50 fs) hole transfer from MoS_2_ to WS_2_, which has excellent potential for various applications, including optoelectronics, light harvesting, and many more [92]. The limit of indirect excitons is generally restricted to low temperatures despite extensive study on indirect excitons in semiconductor vdWHs based on group III–V and II–VI elements, e.g., AlA/GaA-linked quantum wells [93]. Moreover, Figure 4h demonstrates the formation of a coupled quantum well in the vdW MoS_2_/*h*-BN heterostructures illustrated in Figure 4g. Indirect excitons were discovered by Calman et al. at room temperature [94]. Strikingly, the lifespan of indirect excitons in MoS_2_/*h*-BN vdWHs was longer than direct excitons in monolayer MoS_2_.

### 3.3. Energy-Band Alignments

**Figure 5:**
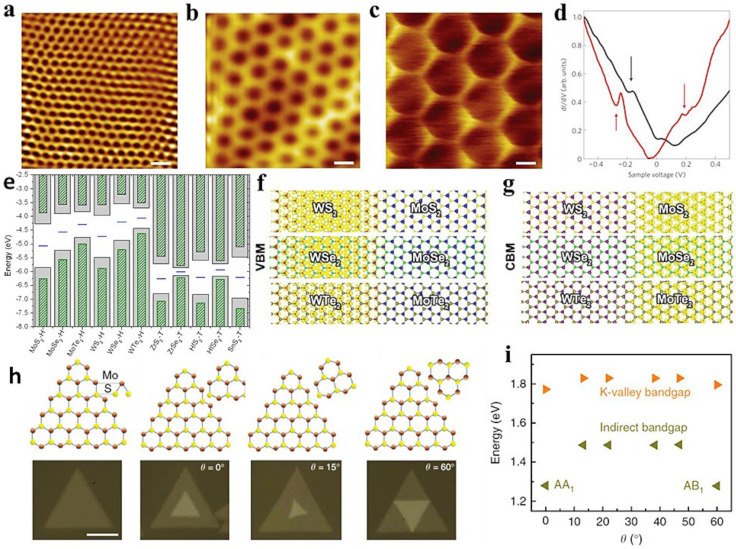
STM topographical photographs at (**a**) 2.4 nm, (**b**) 6.0 nm, and (**c**) 11.5 nm, (**d**) The arrows in the experimental dI/dV curves stipulate the dips at two different moiré wavelengths, 9.0 nm (black) and 13.4 nm (red). The black curve shows a dip energy of 0.28 eV, whereas the red curve shows a dip energy of 0.22 eV from the Dirac point respectively. Further, the valence band exhibits much deeper dips than the conduction band, which indicates different relative strengths. Adapted with permission from Ref. [79]. Copyright 2012, Nature Publishing Group. (**e**) Visualization of band alignment for monolayer semiconducting TMDs as well as monolayer SnS_2_. Adapted with permission from Ref. [104]. Copyright 2013, American Institute of Physics. (**f**) VB maximum and (**g**) CB minimum charge densities in monolayer WX_2_/MoX_2_ (X  =  S, Se, and Te) lateral heterostructures with similar X sites. Adapted with permission from Ref. [102]. Copyright 2013, American Institute of Physics. (**h**) Optical photographs of mono- and bilayer MoS_2_ at certain twisting angles. (**i**) Display of energy values evaluated from Kohn–Sham K-valley indirect bandgaps (green) and direct bandgaps (orange). Adapted with permission from Ref. [105]. Copyright 2014, Nature Publishing Group.

The electronic orbital extends to each other in vdWHs despite the faint interactions among layers at the interface that affect the band structure in every layer [96,97]. A 2D stacked vdW layered structure can exhibit significant interaction between interlayers, producing unique physical characteristics. Because of Dirac electrons’ linear dispersion, graphene has a high carrier mobility of 10^4^ cm^2^/Vs [98]. Still, zero bandgaps of graphene limit its applications in transistors [99,100]. In contrast, graphene-based vdWHs have pioneered several growing research topics, which may compensate for the graphene’s zero bandgaps. For example, controlled growth of graphene layers over *h*-BN can induce arbitrary rotational orientation between the lattices of individual components of the device. Due to the higher lattice constant of *h*-BN and this rotation of lattices, topographic moiré patterns are created as shown in Figure 5a–c [79]. In addition, as displayed in Figure 5d, the moiré pattern functions as a faint periodic potential and thereby producing a new set of Dirac points [79]. This result implies the possibility of controlling graphene’s electronic structures by stacking it over *h*-BN layers. In the case of a new Dirac point, the lattice mismatch among *h*-BN and neighboring graphene layers can tune the density of states in the graphene layer.

Similarly, it suggests that stacking various TMDs can change the electronic structure. Several research groups have theoretically predicted and demonstrated experimentally that vdWHs based on monolayer MoA_2_/WA_2_ (A = S, Se, and/or Te) exhibit type-II band alignments (Figure 5e) [101,102]. Optically active conduction-band (CB) minimum and valence-band (VB) maximum sites are limited to opposing layers, and the lowest-energy excitons can split spatially (Figure 5f–g) [102]. This is promising in some applications, such as optoelectronics and solar-energy conversion. The type-II band alignment in 2D MoS_2_/WSe_2_-based heterostructure was illustrated by Li et al. with 0.76 eV as the conduction offset and a 0.83 eV valence-band offset [103]. In contrast, according to Cho et al., monolayer n-type MX_2_ (where X = Se/Te; M = Mo/W)-based and p-type MX_2_ (where X = S/Se; M = Zr/Hf)-based vdWHs have synergistic coupling that offer broken gap junctions with promising tunneling efficiencies [104]. Such features are of great significance in low-power logic devices. Although 2D MX_2_ (M = Mo/W; X = S/Se) monolayer TMDs are direct-bandgap semiconductors, they change to indirect-bandgap semiconductors as the layers increase. It is due to the non-negligible transformation of the Γ-point to an intermediate state (Γ-Q) [105]. Furthermore, electric and electronic characteristics can be modified for stacked bilayer homostructures by varying the interlayer spacing or twisting the layers. Wang et al. developed MoS_2_ bilayers and found that the size of indirect bandgap changed noticeably according to twist angles, as shown in Figure 5h [105]. Furthermore, it shows that the most significant red shift was observed for AB and AA stacking of layers. In contrast, the red shift was found to be drastically smaller or negligible in the case of other angles (Figure 5i). Here, “AB” suggests the configuration when the S atoms of the top layer are on the S atoms of the bottom layers, whereas “AA” is when the S atoms are on top of the Mo atoms of the bottom layer. It is worth-mentioning that exciton dynamics, Raman spectroscopy, absorption spectra, and PL are optimal approaches for probing optical characteristics of vdWHs because of their efficiency, accuracy, and nondestructive observations [106,107].

**Figure 6:**
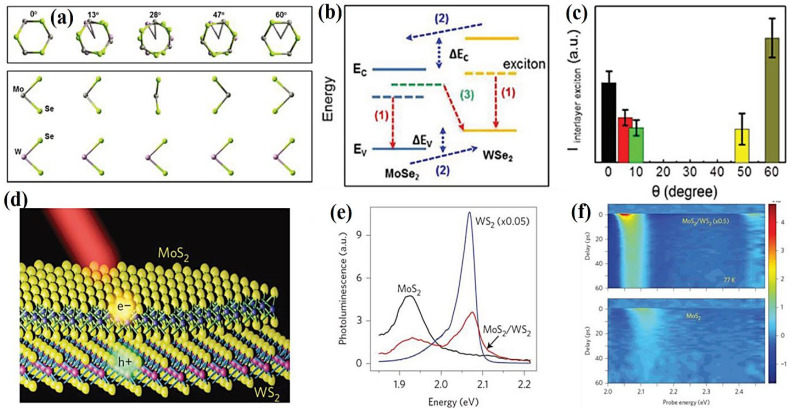
(**a**) Representation of front and lateral views of MoS_2_/WSe_2_ heterostructures at varying twist angles. (**b**) Photoexcited excitonic band alignment of MoSe_2_/WSe_2_ heterostructures. (**c**) Interlayer exciton peak intensity as a function of twist angle. Adapted with permission from Ref. [108]. Copyright 2017, American Chemical Society. (**d**) Atomistic representation of heterostructures based on MoS_2_/WS_2_. (**e**) PL spectrum of isolated MoS_2_, WS_2_, and heterostructures based on MoS_2_/WS_2_. (**f**) 2D plots of transient absorption spectra from a MoS_2_/WS_2_ heterostructure at 77 K(top); and an isolated MoS_2_ monolayer (down) upon excitation of the MoS_2_ A-exciton transitions. Probe photon energy, probe-pump time delay, and transient absorption signal are shown by a horizontal axis, a vertical axis, and a color scale, respectively. Whereas the pump-induced decrease in absorption is indicated by positive signals. Adapted with permission from Ref. [92]. Copyright 2014, Nature Publishing Group.

The production of monolayer WSe_2_ and MoS_2_-based vdWH was explained by Javey et al., who demonstrated a distinct excitonic PL peak at 1.55 eV lower than that of WSe_2_ (1.64 eV) and MoS_2_ (1.87 eV) monolayers [91]. Moreover, they discovered that adding dielectric *h*-BN layers to the vdW gap can alter interlayer coupling. Shin et al. also examined the interlayer coupling between MX_2_-based vdWHs at distinct twist angles [108]. At 0°, the upper layer’s Se-atoms were above the Se-atoms of the underneath layer (Figure 6a). At 60°, the metal atoms of the underneath layer were just below the Se-atoms of the upper layer. Further, PL excitonic peaks were observed at one order of magnitude smaller and slightly red shifted compared to other materials. The red shift of vdWH may have been due to the variations in band structure, and the PL quenching in hetero-bilayer could have been driven by a reduction in PL quantum yield in bilayer systems. Based on band alignment, an observed peak at 1.35 eV in Figure 6b can be ascribed to interlayer excitons. In particular, *d*-orbitals of W and Mo dominated the energy levels of WSe_2_ and MoSe_2_. It can also be observed that the VB and CB of MoSe_2_ were at lower energies than those of WS_2_ because the energy of the 4d orbital of Mo is lower than that of the 5d orbital of W. This causes the type-II band to align between hetero-bilayer systems. When exposed to light, individual WSe_2_ and MoSe_2_ observe exciton formation. As the excitons are located between the CB and VB in each layer, they have lower energy levels than unbound electrons and holes. The photoinduced electrons and holes are subsequently separated and transferred to CB of MoSe_2_ and VB of WSe_2_. Hence, the holes in WSe_2_ VB and electrons in MoSe_2_ CB recombine to generate excitons, consequently leading to interlayer exciton emission. Furthermore, they discovered that PL was maximum at 0° and 60° twist angles during the interlayer exciton emission and diminished at other twist angles (Figure 6c). This shows that the hetero-bilayer system has a highly symmetrical stacking architecture with robust coupling among the layers at twist angles of 0° and 60°. This leads to excellent charge-transfer efficiency due to the short distance between layers, resulting in increased PL intensity. This finding opens ways for modulating the optical characteristics of vdWHs in intricate aspects. Wang et al. employed femtosecond pump-probe and PL mapping spectroscopy in a photoinduced monolayer MoS_2_/WS_2_ heterojunction and demonstrated ultrafast charge transport (Figure 6d) [92]. They identified a significant quenching action of PL spectra at heterojunction compared to the single-layered material (Figure 6e), indicating the high interlayer charge transfer. Following that, they examined the transient absorption spectra of the proposed device in the range of 2.0 to 2.5 eV (Figure 6f) and discovered a hole-transfer time (from MoS_2_ to WS_2_ layer) of 50 fs. Such rapid charge transfer in the proposed vdWHs can be used for efficient solar-energy conversion and high-performance photodetectors.

## 4. Fabrication Routes

### 4.1. Mechanical Transfer

**Figure 7:**
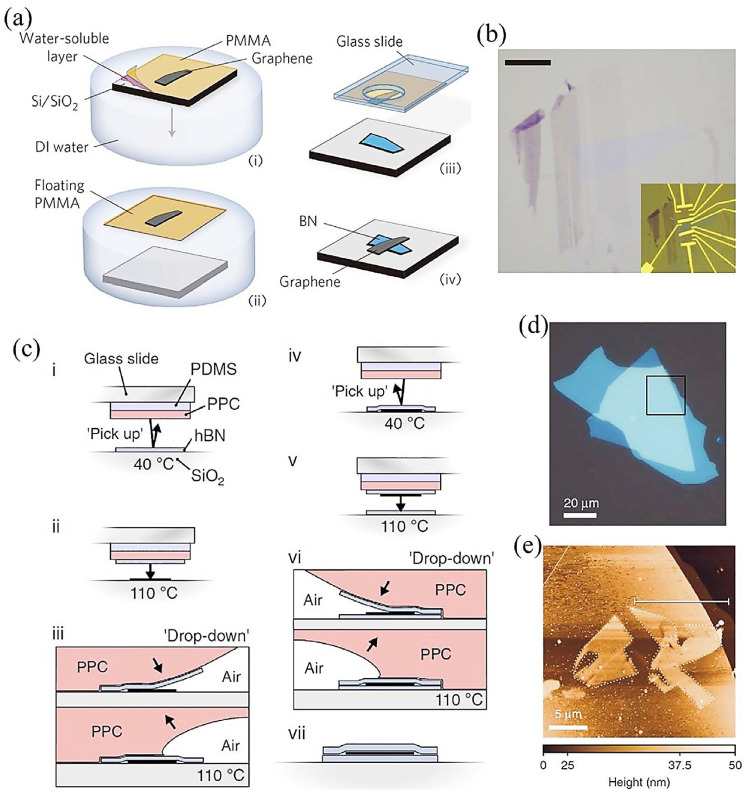
(**a**) Graphic representation of transfer approach utilized to produce devices using graphene on *h*-BN. (**b**) Optical photograph for graphene on *h*-BN (after performing the transfer). The inset shows electrical connections. Adapted with permission from Ref. [70]. Copyright 2010, Nature Publishing Group. (**c**) Schematics of pick-up and drop-down flow process for assembly of 2D heterostructures showing that flakes can be dropped-down and picked-up at the preferable positions. (**d**) Optical microscopic photograph of a staked graphene sandwiched among *h*-BN flakes after the procedure shown in (**c**). (**e**) The AFM of the stacks show no optically visible blisters of contaminations. Adapted with permission from Ref. [110]. Copyright 2016, Nature Publishing Group.

For various 2D materials, wet- and dry-transfer procedures are commonly employed as the mechanical fabrication routes. For example, Dean et al. explained the mechanical wet-transfer method to develop mono- and bilayer graphene over *h*-BN substrates [70]. As indicated in Figure 7a–e, a polymer film comprising a water-soluble layer and a handling layer made of poly(methyl methacrylate) (PMMA) was initially used to translate the graphene. The water-soluble layers get dissolved when immersed in deionized water, which leaves graphene/PMMA layers on top. A micro-manipulator attached to an optical microscope was utilized to position the graphene/PMMA membrane against the target *h*-BN layer after being translated on a glass slide (Figure 7a(ii)). The substrate was rinsed with acetone in subsequent stages to remove the remaining PMMA and heat treated at 110 °C to remove the adsorbed water (Figure 7a(iii–iv)), thus improving the contact between the layers. Optical images of graphene/*h*-BN created by using this mechanical wet-transfer approach are shown in Figure 7b. Furthermore, simply repeating the previously mentioned steps enables the fabrication of complex heterostructures. However, the residues on the surface could impede the transport procedure. Therefore, anticipating the robust vdW interaction among 2D materials, improved pick-and-lift approaches have been established to facilitate the complicated multilayer stacking with impurity-free interfaces [109,110]. A polypropylene carbonate (PPC)/polydimethylsiloxane (PDMS) stamp was utilized to place a desired *h*-BN flake on a SiO_2_ substrate at 40 °C (Figure 7c(i)). Subsequently, *h*-BN was translated to a different substrate at 110 °C (Figure 7c(ii,iii)). Moreover, a subsequent stack can be selected and successively placed onto an underlying *h*-BN substrate, as shown in Figure 7c(iv–vii). Compared to earlier pick-and-drop strategies, the advantage of the vdW pick-and-drop approach is that there is no interaction between the interface and any polymer during transfer. *h*-BN/Graphene/*h*-BN vdWHs assembled through this kind of growth are presented in Figure 7d,e. In addition, AFM analysis, shown in Figure 7e, demonstrated that the graphene layer is completely enclosed among neighboring *h*-BN layers with no prominent blisters. Moreover, several studies have been performed on comparable transfer methods, even with minor alterations in stamp preparation [111]. For instance, as an alternative print, Xu et al. produced a graphene-sandwiched heterostructure (*h*-BN/graphene/*h*-BN) through polycarbonate (PC)/PDMS [111]. It is critical to note that some essential principles, such as the purity of stack, time required for transfer, and simplicity of operation, should be considered while evaluating alternative transfer approaches [112].

### 4.2. Chemical Synthesis

Even though challenging, mechanical transfer procedures show enormous potential in fabricating unique vdWHs using 2D material that incites astonishing physical phenomena and imparts distinct features. Still, such a procedure has scalability constraints. Some alternative strategies, such as the bottom-up approach, have been established to overcome limitations and ensure scalability. The atomically flat surface and the absence of dangling bonds make the graphene an excellent template for other 2D materials and have been used to build several vdWHs [113,114,115]. Compared with manual stacking, direct stacking of 2D vdWHs allows simultaneously automated (machine-driven) manufacturing of several layers. Direct-growth 2D vdWHs may be produced using several methods, including single-step in situ fabrication, physical epitaxy (epitaxial growth), and CVD.

Furthermore, graphene-based vdWHs are helpful in place of various electrical applications owing to fundamental aspects such as minor-resistivity connection originating from semimetal/semiconducting interfaces. For example, graphene film was initially developed using a Cu-foil (graphene/Cu) via the liquid precursor CVD technique. Subsequently, ammonia borane (NH_3_-BH_3_) was utilized to create h-BN layers on graphene/Cu film produced in the previous step. Furthermore, a two-step CVD route was applied by Liu et al. [116] for the growth of h-BN over graphene. In addition, graphene can also be adapted as an epitaxial substrate for TMDs. By using CVD at lower temperature conditions (400 °C), Shi et al., for instance, reported a modest method for producing MoS_2_/graphene vdWHs [115].

**Figure 8:**
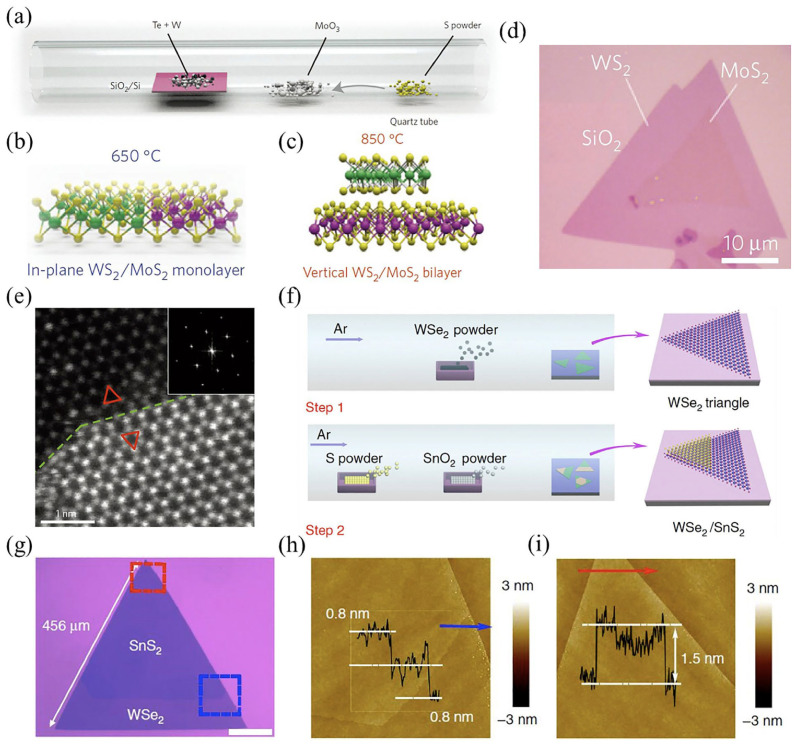
Depiction of synthesis mechanism of vdWHs: (**a**) demonstration of WS_2_/MoS_2_ heterostructure procedure. (**b**,**c**) In-plane and vertical stacking of WS_2_ (650 °C) and MoS_2_ (850 °C) monolayers to grow (in-plane/vertical) heterostructures. (**d**) Display of optical photograph for an in-plane WS_2_/MoS_2_ heterostructure on an SiO_2_ substrate. (**e**) Z-contrast image of the step edge of WS_2_/MoS_2_ bilayer on SiO_2_ substrate. The green dashed boundary implies the edge of the step, while the two triangles show the orientation of the MoS_2_ (top) and WS_2_ (bottom) layers. Adapted with permission from Ref. [132]. Copyright 2014, Nature Publishing Group. (**f**) Pictorial demonstration of dual-step vapor epitaxy fabrication of WSe_2_/SnS_2_ vdWHs. (**g**) Optical photograph of a stacked triangular flake of 1L SnS_2_/1L WSe_2_. (**h**) AFM photograph of the section in (**g**) indicated by the blue rectangle. (**i**) AFM photograph of the section in (**g**) indicated by the red rectangle. Adapted with permission from Ref. [119]. Copyright 2017, Nature Publishing Group.

Moreover, Chen et al. fabricated continuous monolayer MoS_2_ films over graphene as a substrate that provides sufficient information about the quality of MoS_2_ films, which entirely depends on nucleation density and growth time and can be controlled by precisely controlling these parameters [117]. Optics-electronic integration on a single chip has much potential owing to the wafer-scale development of vdWHs. Furthermore, it should be noted that the characteristics of resultant vdWHs were influenced by the quality of the substrate onto which the graphene flake was transferred [118]. Because MoS_2_ and WS_2_ grow sequentially due to nucleation variations and growth rates, vertical heterostructures (WS_2_/MoS_2_) commonly grow at 850 °C compared to in-plane structures (Figure 8a–c). An optical photograph of an in-plane WS_2_/MoS_2_ heterostructure on an SiO_2_ substrate is presented in Figure 8d, whereas Figure 8e shows a Z-contrast image of the step edge of a WS_2_/MoS_2_ bilayer on SiO_2_ substrate. In addition, the direct vdW epitaxial growth of the WSe_2_/SnS_2_-based vertical bilayer p–n junction with lateral diameters close to the millimeter scale has also been reported [119]. Using WSe_2_ powder as a precursor, the underlying WSe_2_ monolayer was produced at 1100 °C, whereas above this temperature, an SnS_2_ layer was produced with SnO_2_ with S-powders as precursors and the Ar as the carrier gas (Figure 8f). Furthermore, the AFM images presented in Figure 8h–i substantiate that the bilayer vdWH, which is shown in Figure 8g, has one layer of WSe_2_ and one layer of SnS_2_. Various vdWHs, such as MoTe_2_/Wse_2_ [120], GaSe/MoSe_2_ [121], GaSe/MoS_2_ [122], Sb_2_Te_3_/MoS_2_ [123], and many others, have been fabricated using a direct CVD method. Aside from the above-mentioned CVD procedures, several other approaches, such as metal-organic (MO)-CVD [124] and molecular beam epitaxy (MBE) [125,126], can be employed to build vdW 2D heterostructures directly. MOCVD uses organometallic compounds because they vaporize at a higher pressure than metallic elements [127]. After vaporization, these compounds are transferred to the growth chamber via a carrier gas. Since MOCVD uses organometallic precursors that degrade below 400 °C, it is possible to perform the synthesis at low temperatures. It also indicates that organometallic compounds can be fine-tuned in a bubbler modifier that controls the flow rate of the carrier gas (typically H_2_ or N_2_). However, a thorough study of the purity and decomposition pathway of the precursors is essential to produce carbon-free films under the required growth conditions [128,129]. MOCVD can be performed in cold wall reactors and horizontal hot wall reactors, unlike other techniques, such as powder-based chemical-vapor deposition (P-CVD). Cold wall reactors minimize unwanted gas-phase reactions by limiting the region of thermal heating near the substrate. It is worth mentioning that the MOCVD technique is much more universally applied to 2D heterostructures, including group III oxides [130] and group III nitrides [34]. The importance of defects was described by Azizi et al. via MOCVD to fabricate freestanding WSe_2_/graphene vdWHs [124]. They employed CVD to create a thin graphene film on a Cu-substrate, which was then transferred to a gold TEM grid. To create a monolayer WSe_2_, an Au grid and graphene sheet were mounted inside an MOCVD device. The growth was carried out in a hydrogen atmosphere using dimethyl selenide as selenium and tungsten hexacarbonyl (W(CO)_6_) as a tungsten precursor. 

A further potential method is an ultrahigh vacuum (UHV) –MBE, which is a common technique employed for II–VI and III–V semiconductors. TaSe_2_/MoSe_2_ and TaSe_2_/HfSe_2_ vdWHs were produced by Tsoutsou et al. by utilizing UHV–MBE, and the resulting structures displayed good structural stability [131]. The direct-growth approach is generally considered the most promising strategy to realize scalable production of various vdW 2D heterostructures. Furthermore, it provides absolute control over the thickness and stoichiometry of layers and prevents exposure to ambient contaminants that harm the device’s performance. Additionally, controlled development of vdW 2D heterostructures continues to be a significant challenge despite significant efforts and cutting-edge technologies over the past 10 years. Specific factors that rely on growth conditions, such as crystallinity, homogeneity, and thickness, should be considered. Nevertheless, each 2D material has its own requirements and has a significant impact on interface properties. Additionally, ideal epitaxial growth can only be achieved between materials with perfectly matched lattices.

## 5. 2D Heterostructure-Based Optoelectronic Devices

### 5.1. Photodetectors

The development of highly effective photodetectors with a broad optical response is made possible by the rapid growth of 2D materials with specific optoelectronic and electrical properties [5,133]. As described earlier, the dangling bond-free nature of 2D materials allows for the simple fabrication of multidimensional heterostructures [134,135]. The fabrication of 2D vdWHs is a potential strategy for realizing flexible photodetector devices with diverse topologies [136,137]. Based on varied material stacking, four primary forms of 2D vdWHs for photodetectors are established. The first one is based on graphene/2D-semiconductor/graphene (G/2D-SC/G) heterostructures in which graphene serves as the contact layer and 2D semiconductors serve as the photoactive layer. The spectral detection range in this 2D vdWH is primarily dictated by various bandgap energies of 2D semiconductors. These patterns are deemed advantageous for minimizing contact resistances and accelerating the split-up of photogenic carrier pairs to yield highly effective photocurrent and increased photo-reaction speed.

**Figure 9:**
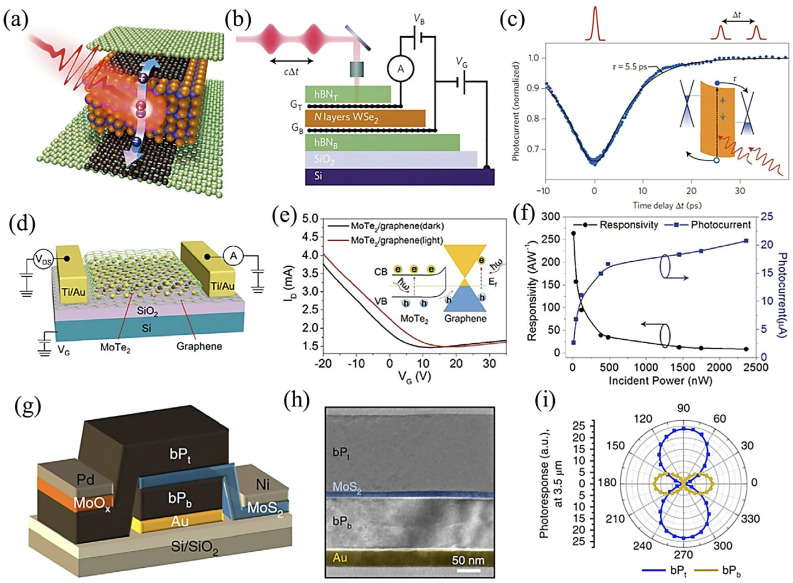
(**a**) Atomistic representation of photoexcited carrier dynamics in an *h*-BN/G/WSe_2_/G/*h*-BN heterostructure. Excitons are produced, separated, and delivered to graphene electrodes under pulsed-laser excitation. (**b**) Demonstration of time-resolved photocurrent-analysis method. (**c**) Photocurrent time-delay behavior demonstrates a light response-time constant of 5.5 ps. Adapted with permission from Ref. [138]. Copyright 2016, Nature Publishing Group. (**d**) A schematic representation of photodetector based on MoTe_2_/graphene. (**e**) Charge-transfer behavior of the device (with and without illumination). (**f**) The behavior of photocurrent and photoresponsivity as a function of incident light power at 980 nm. Adapted with permission from Ref. [142]. Copyright 2017 John Wiley & Sons, Ltd. (**g**) Schematic of BP_t_/MoS_2_/BP_b_ vdW heterojunction-based devices. (**h**) Cross-sectional TEM images of polarization-resolved BP/MoS_2_ heterojunction photodetector. (**i**) Photo response of MoS_2_/BP_t_ and MoS_2_/BP_b_ under a linearly polarized laser at λ = 3.5 μm. Adapted with permission from Ref. [141]. Copyright 2018, Nature Publishing Group.

Additionally, by tuning the gate voltage, the work function of graphene can readily be changed to change the photocurrent of the proposed device. A 2D vdWH composed of *h*-BN/G/WSe_2_/G/*h*-BN with an ultrashort photo-response-time constant has been demonstrated. Such heterostructures exhibit an exceptional photodetection performance, which acts as a function of bias voltage and can also be controlled by adjusting the thickness of WSe_2_ flakes [138]. In this case, the encapsulating layer is *h*-BN, the source and drain electrodes are monolayer graphene flakes, and the photoactive material is WSe_2_. Furthermore, graphene improves the light response by increasing the charge-transfer rate (Figure 9a). In the meantime, measurements of the temporal response of *h*-BN/G/WSe_2_/G/*h*-BN photodetector were made using time-resolved photocurrent measurements (Figure 9b). The photocurrent response-time constant of the G/WSe_2_/G photodetector was estimated to be 5.5 ps under sub-picosecond laser stimulation (Figure 9c). In principle, the development of efficient and quick photodetection response depends on intrinsic features, e.g., the atomically narrow charge-extraction channel of the device.

The structural design of the second device is founded on the photogate of vertical vdWHs, where different 2D materials with distinct properties are commonly coupled to provide higher carrier mobility (as the charge-transport layer) and outstanding light absorption (as the light-absorption layer). Graphene has higher carrier mobility and offers superior interface properties in the heterostructure so it can function as a charge-transport layer. On the other end, MoTe_2_ has better light-absorption characteristics and serves as a light-absorption layer. A MoTe_2_/graphene vdWHs-based photodetector was recently reported (Figure 9d) [138]. Upon illumination, the photoexcited carriers in the MoTe_2_ layer were transported easily and rapidly to the graphene layer because of the matching energy-band structure (Figure 9e). By unifying the properties of both materials, vdW heterojunction devices exhibited an exceptional performance under 1064 nm laser stimulation, thus providing a high photo responsivity, an ultrahigh photoconductive gain, and a detectivity (D*) of ~970.82 A/W, 4.69 × 10^8^, and 10^11^ Jones, respectively (Figure 9e,f). This shows that the high mobility and high light absorption of two different 2D materials can successfully be combined in vdWHs to achieve high-performance photodetection.

The third kind of vdWHs, i.e., vdW p–n heterojunctions, has also been studied significantly and is considered the most fundamental and extensively used building block for industrial semiconductor devices. p–n vdWHs may effectively segregate photo-excited carriers and provide photocurrent regardless of the momentum mismatch among the layers. There are two significant benefits to fabricating an atomic vdW p–n junction in this situation: (i) the photoexcited electron–hole pairs created by individual material can be effectively divided by a built-in electric field close to the p–n junction interface, enabling rapid photodetection, and (ii) complementary p–n junction-based photodetectors typically explain the benefits of using two materials in p–n junctions that give rise to different behaviors, such as detection-band complementarity and polarized light-response interaction. Despite the interlayer momentum mismatch, p–n vdW heterojunctions efficiently split up photo-excited carriers and lead to a photocurrent [139]. Chen et al. use ultrafast visible/infrared micro-spectroscopy to investigate the interlayer charge-transport kinetics in MoS_2_/WS_2_ vdWHs [140]. The charge transfers between the MoS_2_ and WS_2_ layers produced an intermediate state of electron–hole pairs with excess energy before creating firmly bound interlayer excitons. The surplus energy in the intermediates makes it easier for them to dissociate, which helps generate the photocurrent. A recently reported BP/MoS_2_ heterostructure composed of mid-wave infrared (MWIR) polarized detectors with “Au” as the contact with the hole and back reflector and “MoS_2_” as the MWIR windows and contact source with the electron. The proposed device displayed a clear room-temperature photo response with excellent performance [141]. Because of BP’s thickness-dependent light-absorption properties, a thick flake of 150 nm was chosen as the light-absorption layer, with an 80% x-polarized incoming laser absorption capacity. At ambient temperature, a 150 nm BP/15 nm MoS_2_ vdWH-based photodetector demonstrated an EQE (external quantum efficiency) of 35% at 2.5–3.5 μm and a specific D* of up to 10^10^ Jones at 3.8 μm, with an R = 0.9 A/W·m. Furthermore, a photodetector based on MoS_2_ sandwiched between two BP layers (BP_t_/MoS_2_/BP_b_) was fabricated for polarization-resolved and bias-dependent photodetection (Figure 9g,h). The photo response of MoS_2_/BP_t_ and MoS_2_/BP_b_ as a function of the polarizer angle under a linearly polarized laser at λ = 3.5 μm is presented in Figure 9i. It illustrates that the proposed device can simultaneously detect two linear polarization components under unpolarized illumination. Following that, BP_t_ crystal orientation of the top BP layer (BP_t_) is perpendicular (out-of-the-axis) to the BP layer at the bottom (BP_b_), and the adjacent MoO*_x_*/Pd layer functions as a hole collector from the BP_t_ layer.

#### Photodetectors in Biomedical Field

**Figure 10:**
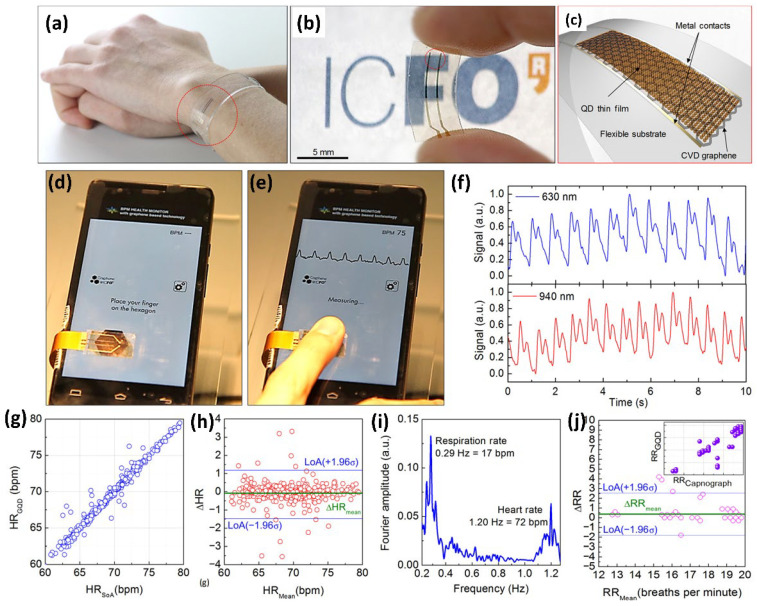
(**a**) Illustration of transparent and flexible GQD photodetector implanted in an HR tracking wristband. (**b**) A zoomed-in view of stretchable photodetector on polyethylene naphthalate substrate with a 1 mm^2^ graphene channel entirely encased in a thin 30 nm-thick coating of PbS QDs. (**c**) Schematic representation of graphene and QD setup on a flexible substrate assembly. Photodetection in graphene/PbS QD heterostructure-based health patches for wearable fitness via HR and RR monitoring: (**d**,**e**) a mobile-phone screen with an adaptable health patch. (**f**) Signals from photoplethysmography (PPG) at 630 and 940 nm. (**g**) Two simultaneous readings of a health patch and a cutting-edge PPG sensor. (**h**) Bland–Altman assessment of the patch (**i**) Fourier transforms of PPG’s. (**j**) Bland–Altman evaluation of the obtained RR. Adapted with permission from Ref. [145]. Copyright 2019, AAAS.

Past years have seen extensive use of TMDs in numerous biomedical applications such as UV-radiation monitors, retinal prostheses, and pulse oximeters [143,144]. In addition to significantly reducing medical costs, wearable photodetectors are essential for health monitoring. In this regard, advanced strategies are required to fabricate devices with greater flexibility, ease of integration, and environmentally stable materials. Two-dimensional materials are an obvious choice for wearable electronics due to their inherent advantages of size-dependent properties, flexibility, and intriguing optoelectronic characteristics. For example, tattoo-like graphene devices record skin hydration, body temperature, and heart rate (HR). Recently, a wearable device was created by Polat et al. by placing light-sensitive lead-sulfide (PbS) quantum dots (QDs) on graphene [145]. Here, graphene functions as a susceptible substrate in connection with QDs, which means that their incorporation in the biomedical sector has excellent potential for sensing over a broad spectrum of wavelengths [146]. Electron–hole pairs in PbS QDs are produced upon illumination, where the holes are transmitted to graphene, and electrons remain confined inside the QDs. Holes transmitted in graphene improve conductivity, thus producing a controlled electrical response. The high sensitivity can be attributed to the long-term electron trapping in QDs leading to an increase in photoconductive gain through different charge carrier productions for an absorbed photon. This inherent gain reduces the necessity for an external amplifier, lowers energy usage, and creates a small device for wearable applications. It can distinguish specific light wavelengths that hemoglobin in blood absorbs and assess volumetric variations in blood vessels connected to HR. Additionally, the wearable electronic device enables the forecast of saturated oxygen levels (SpO_2_) in the blood because light absorption differs from oxygen levels in blood cells. This can also function as HR and respiration rate (RR) indicators on human skin. Surprisingly, the sensor is sensitive enough to use ambient light, which is essential for useful applications in health monitoring. As illustrated in Figure 10a–c, Polat et al. recently reported a novel class of transparent and flexible wearables fabricated of graphene-coupled semiconducting quantum dots (GQD) [145]. They reported numerous prototype wearable devices that noninvasively monitor essential health signals such as HR, RR, and SpO_2_. It demonstrates the utilization of ambient light with minimal power usage. Additionally, near-field communication (NFC) circuit boards with flexible UV-sensitive photodetectors enable wireless data and power transmission between photodetectors and cellphones. This technology opens the door for entirely connected wearables through wireless UV-index probing and empowers the user (Figure 10d–j).

### 5.2. Light-Emitting Diodes

Having the possibility of direct bandgap to the near-infrared (NIR), single-layer TMDs present a promising opportunity for optoelectronic applications and offer promise in the fabrication of LEDs [147,148]. Several studies have demonstrated electroluminescence (EL) in a monolayer MoS_2_-based transistor, possibly due to a similar excited state (i.e., exciton-A) [149]. Since EL emission is restricted to metal contacts in the case of single-layer MoS_2_, QE was limited to 10^−5^. Fabricating a LED based on a p–n diode proved to be a practical approach to increasing EL efficiency. Recently, a dual-gating method efficiently determined the in-plane p–n junctions in monolayer TMDs in which the active area was centered at the depletion region [150,151].

**Figure 11:**
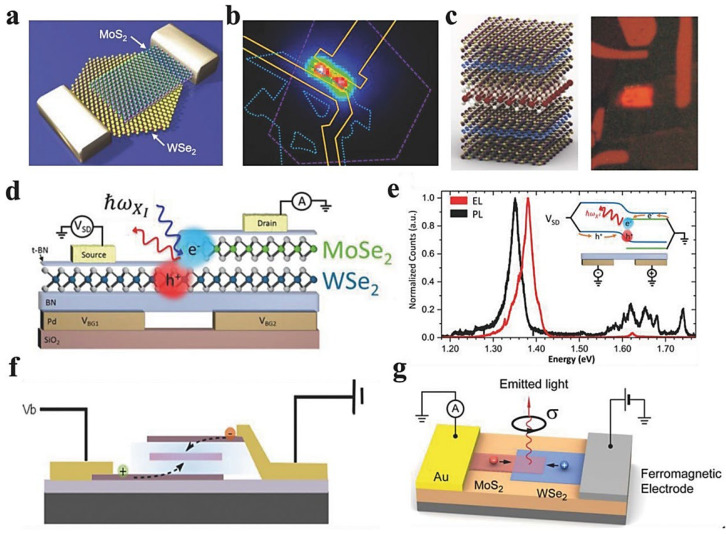
(**a**) Graphical illustration of heterostructure based on WSe_2_/MoS_2_. (**b**) EL spectra (false color) of device using 100 μA injection current. Adapted with permission from Ref. [152]. Copyright 2014, American Chemical Society. (**c**) Atomistic representation of *h*-BN/graphene/2*h*-BN/WS_2_/2*h*-BN/graphene/*h*-BN single-quantum wall heterostructure and optical view of EL using the alike device. Adapted with permission from Ref. [157]. Copyright 2015, Nature Publishing Group. (**d**) Representation of assembly for heterostructure. (**e**) Visualization of PL and EL spectra of heterostructure (inset: display of carrier transportation). Adapted with permission from Ref. [162]. Copyright 2017, American Chemical Society. (**f**) Demonstration of working of LED composed of a vertical heterostructure. Adapted with permission from Ref. [162]. Copyright 2016, American Chemical Society. (**g**) Schematics depiction of the proposed device. Adapted with permission from Ref. [164]. Copyright 2016, American Chemical Society.

On the contrary, vertical vdWHs have emerged in place of effective carrier addition in LEDs because of the wide active area that spans overlapping junctions [152,153]. For example, Duan et al. [152] studied EL characteristics of p–WSe_2_/n–MoS_2_ diodes (Figure 11a). However, after a 3V forward bias, photocurrent demonstrated that the EL signal primarily originated from the overlapped region close to metal electrodes. Only a few electrons could move from MoS_2_ to WSe_2_, but WSe_2_ holes are injected into MoS_2_ when a forward bias was minimal and below a certain threshold. In MoS_2_, radiative recombination was minimal because of the indirect bandgap. Excitons are added into p- and n-type materials when the bias voltage is raised over the threshold. In addition, they could flow through the junction because of the increasing migration of MoS_2_ CB. Whereas radiative recombination in WSe_2_ dominated the EL, it increases linearly with injected current. Remarkably, the fitting of EL spectra using multiple Gaussian functions caused prominent peaks at 546 and 483 nm that corresponded to electron luminescence; these peaks can be used to investigate the interaction among electron-orbital in the lattice of WSe_2_. Notably, the EL peaks of the electrons were found at 546 and 483 nm after data fitting using Gaussian functions. These peaks are significant for interpreting the electron-orbital interaction in WSe_2_ (specifically, the interaction between the electron and its orbital). To create functional stacking of structures for controlled light emission, it is necessary to incorporate tunneling layers into electrode contacts and p–n junctions. This reduces leakage current in stacked arrangements and increases the lifespan of electrons and holes in TMD quantum wells [154,155]. A metal-insulator semiconductor-based tunnel diode made of monolayer WS_2_, multi-layer graphene, and *h*-BN exhibited an excellent QE (~1%) [156]. As shown in Figure 11b (quantum wells), the graphene-based effective LEDs created by Novoselov et al. consisted of conductive layers in which *h*-BN served as tunneling barriers and a variety of TMDs as quantum wells [157]. Advanced current densities and bridged contact resistance enabled luminous LEDs by pumping excitons from graphene electrodes into the TMD layer (Figure 11c). Nevertheless, there was an abrupt shift in the PL spectrum at a specific gate voltage, showing a different peak at 1.90 eV. This shift was produced via the Fermi level of the bottom graphene moving over the CB of MoS_2_, enabling electrons to flow into the quantum well. They also included numerous quantum wells stacked in sequence to enhance the possibility of radiatively recombining the injected carriers. The acquired QE could approach 10%, almost 10-fold greater than planar p–n diodes [150] and, interestingly, 100 times better than Schottky-barrier diodes [149]. In addition, monolayer TMDs had direct bandgap semiconductors consistent with tremendous tunability, making them perfect for atomically thin LEDs. White LEDs offer promising potential in lighting and display because of their elevated brightness at low power consumption and an extended lifespan for efficient operations. Chen et al. engineered a white LED that comprises n-MoS_2_/p-MoS_2_/p-GaN as blue, green, and orange emitters [158]. These heterostructures exposed the capacity to create light sources (atomically thin) with white LEDs, with PL spectra at 481 nm (blue, p-GaN), 525 nm (green, p-MoS_2_), and 642 nm (orange, n-MoS_2_) [158]. Since type-II band alignment is primarily acquired in vdWHs, the excitons pairs can be effectively separated. Furthermore, thermal light emission from graphene [159] and MoS_2_ [159] was reported with improved bright-light emission through the suspended device. However, because of limited interlayer separation, temporally isolated electrons and holes endure significant Coulomb interactions, leading to a tightly bound interlayer exciton (XI) [160]. To investigate the EL characteristics, Xu et al. [161] electrostatically developed a MoSe_2_/WSe_2_ hetero-bilayer-based in-plane p–n diode (Figure 11d). Several peaks in MoSe_2_ and WSe_2_ PL spectra from 1.56 to 1.74 eV indicated intralayer A-excitons and neutral, charged, and localized excitons (Figure 11e). Furthermore, Xu et al. [161] created a heterostructure consisting of stacked graphene layers as electrode materials separated by a thin layer of WSe_2_ with BN barriers, as visualized in Figure 11f [162]. When the external bias was zero, the Fermi level for graphene layers fell within the bandgap limits of WSe_2_. Moreover, the addition of spin from a ferromagnetic electrode was introduced by Kis et al. into a WSe_2_/MoS_2_ monolayer heterostructure [163]. This caused circularly polarized light emission that was controllable by an external magnetic field (Figure 11g).

### 5.3. Photovoltaics

**Figure 12:**
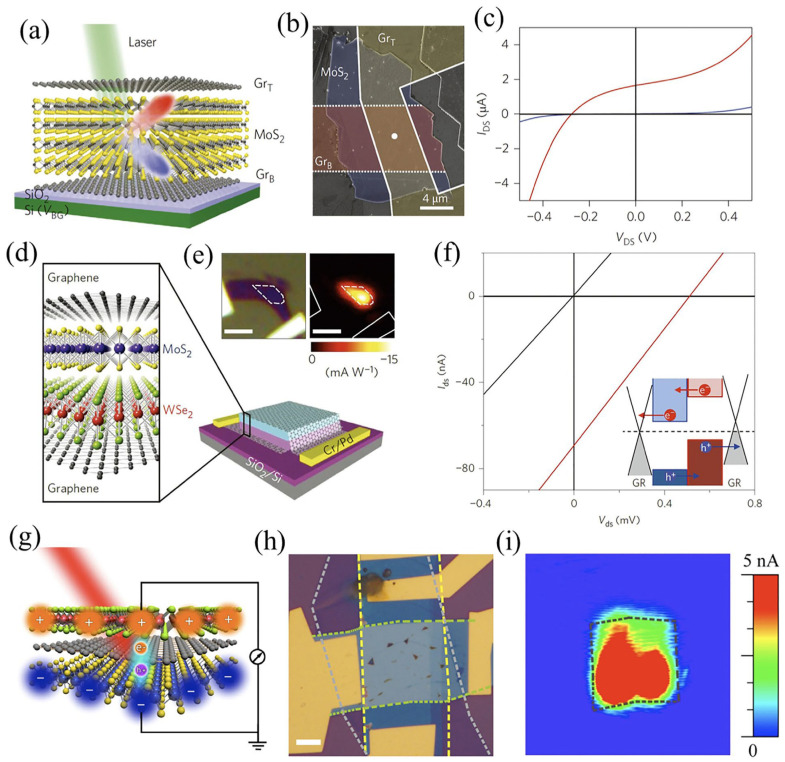
(**a**) Multilayer MoS_2_ sandwiched among graphene electrodes (edge view). (**b**) The graphene layer at the bottom graphene (red), middle (blue), and top MoS_2_ layer (yellow) are each falsely colored in the SEM photograph of the device. (**c**) I–V behavior of the vertical device. Adapted with permission from Ref. [169]. Copyright 2021, Nature Publishing Group. (**d**) Representation of graphene sandwiched between an MoS_2_/WSe_2_ junction. (**e**) Photocurrent map for the corresponding junction (V_ds_ = 0 V) and an optical photograph of a single-layer p–n junction device (1L–1L) sandwiched between a graphene electrode. (**f**) I–V curves of the device publicized in the figure restrained in the dark (black) and under 532 nm laser excitation (red). Adapted with permission from Ref. [153]. Copyright 2014, Nature Publishing Group. (**g**) Illustration of photodetector based on p–g–n heterostructure. (**h**) Optical microscopic view of the device. (**i**) Photocurrent mapping from the narrow p–g–n junction at V_ds_ = 0 V. Adapted with permission from Ref. [171]. Copyright 2016, American Chemical Society.

Attempts have been made to simultaneously explore the conversion of light energy by means of advancing vdWH-based photodetectors and LEDs. As reported by Jariwala et al., 2D vdWHs have a power-conversion efficiency (PCE) of greater than 25% [163], which is equivalent to standard solar cells. Using vdW 2D heterojunctions that consist of higher-quality hetero interfaces allows for excellent charge separation when exposed to photo-excitation; this could result in more significant potential for ultrathin and lightweight photovoltaic applications. Furthermore, combining monolayer 2D materials, conventional semiconductors, and direct bandgap materials can produce effective single junctions. Following this, the fabrication of a photovoltaic device described by Sanchez et al. consisting of a MoS_2_/p-Si monolayer-based hybrid p–n heterojunction demonstrated a broad-spectrum response and an EQE of more than 4% [165]. Moreover, studies have been performed on chemical and/or electrostatic doping in the same 2D flakes to design new homojunctions [166,167]. At 0.14 V open-circuit voltage (V_oc_), Li et al. demonstrated a NIR photovoltaic outcome in a p–n BP homogenous junction doped with Al when irradiated with a 1550 nm laser. The proposed photovoltaic device showed a PEC of 0.66% [168]. Pospischil et al. also investigated an in-plane p–n homojunction based on a WSe_2_ monolayer using electrostatic doping under local gate control and demonstrated a PCE of 0.5% in addition to a fill factor of 0.5 [166]. A substitute method for building efficient and adaptable solar devices is to use vdWHs fabricated by stacked 2D crystals. The evaluation of photovoltaic impact was provided by Brignell et al. for graphene/WS_2_ or MoS_2_/graphene stacks realized via the dry-transfer technique [26]. A significant photocurrent of 1 A has been detected in vertical graphene/WS_2_/graphene heterostructures by providing a doping effect to two distinct layers of graphene and tuning the Fermi levels appropriately. Furthermore, placing Au nanospheres over graphene/TMDs/vdWH causes maximized light absorption of the photoactive layer, leading to a 10-fold increase in photocurrent. Moreover, Yu et al. reported a better photovoltaic effect in vertical stacks of graphene/MoS_2_/metal and graphene/MoS_2_/graphene, attaining 0.3 V of V_oc_, 50 s of rapid temporal response, and 2 μA short-circuit current (Figure 12a–c) [169]. As a dual-gate device, a graphene/MoS_2_/graphene vertical heterostructure can also be constructed, which allows the external electric field to regulate the amplitude of the output photocurrent. They show significant promise for solar-energy applications due to their ability to segregate photo-excited carriers effectively. The photocurrent is produced upon diffusion of relaxed electron holes to connections. Furchi et al. used monolayers of MoS_2_ (n-type) and WSe_2_ (p-type) to build a vdW p–n heterojunction. Upon illumination under white light, they observed a substantial photovoltaic effect with a 0.5 fill factor and 0.2% PCE [170]. The lowest-energy CB states can be found in MoS_2_ layers, whereas the highest-energy VB states can be found in WSe_2_ layers, forming a type-II heterojunction. In addition, WSe_2_ and MoS_2_ produce exciton pairs when they absorb photons from incoming light. Moreover, charge transfer happens through the heterojunction because of type-II offsets of CB and VB after the relaxation of photogenerated carriers. By introducing graphene electrodes directly to the top and bottom of a vertical MoS_2_/WSe_2_ junction, Lee et al. (Figure 12d) developed a graphene-sandwiched vdWH. Instead of diffusion, this method enables more effective vertical charge transfer to collect the carriers. The photocurrent can be acquired where the graphene electrode and p–n junction overlap, according to photocurrent mapping (Figure 12e). Figure 12f shows 70 nA ISC under a 532 nm laser (920 Wcm^−2^), demonstrating the lateral device’s superior carrier -collecting performance. Further structural engineering of vertical vdW p–n heterojunctions can result in a broader spectrum of absorption spectra. Long et al. [171] employed MoS_2_/graphene/WSe_2_ (p–g–n) vdWH to produce a broadband photovoltaic detector (Figure 12g). The optical image at a scale bar of 5 μm is presented in Figure 12h, where yellow, light gray, and green dashed lines highlight MoS_2_, graphene, and WSe_2,_ respectively. This indicates that the photocurrent originates from the narrow p–g–n junction, as presented in the photocurrent mapping (Figure 12i), at V_ds_ = 0 V when irradiated with an 830 nm laser (with a power of ~20.5 W). Hence, a noticeable photo response occurs at the touched area of WSe_2_, graphene, and MoS_2_.

### 5.4. Acoustic Cavities

**Figure 13:**
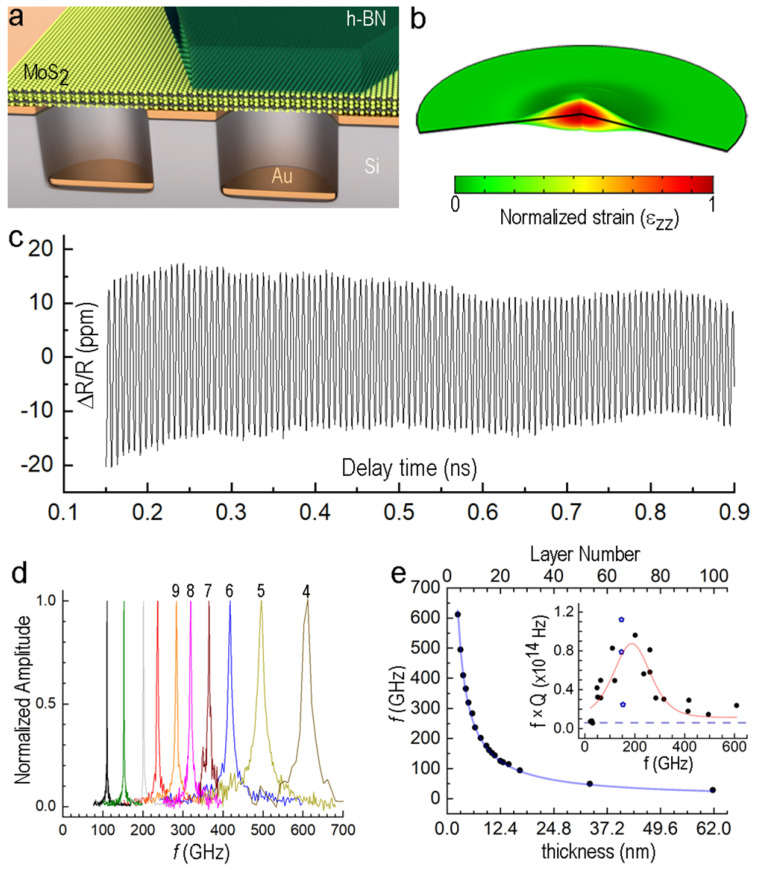
2D heterostructure-based acoustic cavities: (**a**) Illustration of MoS_2_/*h*-BN heterostructure-based acoustic cavities. (**b**) Finite-element modeling simulation of the normalized mechanical strain of suspended MoS_2_. (**c**) Time-varying reflectance of suspended MoS_2_. (**d**) Time-dependent reflectivity spectra from various MoS_2_ cavities calculated via FFT. (**e**) The quantity of MoS_2_ determines the frequency of various MoS_2_ acoustic cavities. Adapted with permission from Ref. [172]. Copyright 2021, Nature Publishing Group.

The use of 2D materials in acoustic cavities is one of the most promising research areas [172,173]. At the same time, the heterostructures of such materials exhibit superior physical properties and provide robust responses [173,174]. Zalalutdinov et al. demonstrated a room-temperature longitudinal acoustic phonon lifetime in *h*-BN- and MoS_2_-based vdWHs (Figure 13) [173]. It exhibited a frequency range of 50–600 GHz, phonon lifetime of 2 ns at 100 GHz in the case of MoS_2_, quality-factor index (*f × Q*) > 10^14^, and coupling power (i.e., 47 GHz) for acoustic cavities (Γ). Compared to the *h*-BN phonon lifetime (0.2 ns), these findings describe significant promise for applying 2D materials at a broad spectrum of frequencies from GHz to THz. These results stimulate advanced acoustic cavity-design engineering with phonon-mediated signals and provide a further degree of freedom by manipulating the quantum nature of phonons using 2D vdWH.

## 6. Challenges and Prospects

Combining different materials and controlling their properties opens up new possibilities for exploring their potential in various applications. However, more research is needed to improve their performance and overcome the challenges associated with implementing these structures. The investigation of innovative 2D materials, for example, graphene, TMDs, BP, MXenes, and others, has led to advances in functional information devices. From theoretical design to device configuration, as well as in the preparation of materials and integration techniques, there has been significant progress in developing 2D material-based optoelectronic devices. This includes sensors, frequency converters, plasmon-generator modulators, ultrafast lasers, and photodetectors. Due to their vast potential, 2D materials can effectively improve the performance of optoelectronic devices and have gained substantial consideration in this field. This article summarizes the properties of current 2D materials and their applications in the field of optoelectronics.

In recent years, 2D vdW structures have been the focus of increased research, but only a few structures have been synthesized experimentally. Derivatives of two naturally occurring vdW materials, molybdenite and graphite, produced through exfoliation and synthesis, have fascinated the scientific community. In addition, to improve the performance of 2D materials, modifications in the fabrication and pre-/post-processing approaches are essential. Techniques such as doping, chemical modification, and electrostatic control can also provide the maximum potential of a material while overcoming possible limitations. In particular, paying attention to the complexities associated with producing high-quality 2D heterostructures with significant phase purity and precise controllability is important. In this respect, it is essential to highlight synthesis methods. Two-dimensional materials can be obtained in several ways, including liquid-phase exfoliation (LPE), mechanical exfoliation (ME), MOCVD, and CVD. However, there are limitations to most of these approaches that prevent further research and application of 2D materials. For instance, many fundamental studies and characterization tools have widely been used to study 2D materials produced by dry mechanical exfoliation. Nonetheless, the lateral dimensions of the layers are usually in micrometers, and it is tricky to cope with the thickness. In contrast, it is possible to produce wafer-scale monolayers using CVD, but the polycrystallinity of the material leads to higher defect density and produces low-quality sheets. To overcome this, CVD systems can be modified to have a better vacuum, more uniform gas flow/pulse, and other reactors in parallel to controlling chemical interactions between precursors in the vapor phase. In addition, the uppermost surface of metal substrates (such as Cu, Ge, W, etc.) must be fabricated first from single-crystal substrates. Even though LPE is scalable for numerous applications, it often lacks high-quality flakes and produces small flakes without precise control. Concerning morphological characterizations, SEM, TEM, STM, and spectroscopic techniques (e.g., Raman, FTIR, etc.) are typically used to analyze 2D materials but specific imaging or characterization techniques are also crucial. However, the effect of lateral thickness, number of layers, doping, modulation of charge carriers, and optimization of protocols to tune electronic and optoelectronic properties needed to be thoroughly investigated to obtain controlled performance and quality. Additionally, it is vital to examine defects and doping at the atomic scale and understand how they affect the band structures of 2D materials.

Importantly, there is also a lack of detailed underlying physics that can be used to analyze carrier transport in vdWHs, which impedes the development of high-performance vdWH devices. Equivalently, band alignment is a fundamental concept and pivotal to understand when describing device physics. Depending on the material, band alignment can be type I, type II, or type III, and it is possible to assess band alignment analytically (e.g., via PL measurements) or theoretically. Significantly, due to the large and expanding family of 2D materials and the infinite number of possible heterostructures, a systematic method for estimating the band alignment of vdWHs is needed. Understanding the physics and adjusting/tuning the coupling between layers and interlayer alignment is also tricky. These variables drastically affect the band alignment and, therefore, the applications of vdWHs. Such technological constraints make it difficult for researchers to overcome the current limitations and use the physical properties of vdW structures for practical purposes. Besides the issues mentioned above, the environmental stability of 2D heterostructures is another critical concern that needs to be addressed to achieve outstanding potential for real-world applications. This includes thermal decomposition, chemical stability, oxidative/environmental stability, and mechanical stability (especially for flexible optoelectronics). Significant experimental/theoretical studies have been performed [175]; however, it needs to be addressed meticulously. In conclusion, and as far as practical aspects are concerned, there are still many obstacles to overcome, but these obstacles could open up many doors. In addition, based on the current state of development, we present here some key directions and our subjective assessment of the development trend:

1. Functional optoelectronic devices based on vdWHs are designed for various optoelectronic applications, including ultrafast lasers, high-speed modulators, ultrasensitive sensors, ultrahigh-responsivity photodetectors, and ultralow damping plasmonics. Most research has focused on graphene, but TMDs and black phosphorus have interesting band structures and spectral responses. Moreover, newly discovered family of 2D materials i.e., MXenes can prove to a better replacement.
2. The possibility of using new metamaterials in optoelectronics, such as perovskites and topological insulators, should be investigated.
3. Develop innovative methods to prepare 2D materials, such as developing functional inks.
4. Research into new optical arrangements and systems for 2D optoelectronic devices, including phonon lasers and extraordinary spots, should be conducted.
5. Incorporating artificial intelligence (AI) into 2D material research to create brain-like devices, and synergistic integration with the Internet of Things (IoT) can lead to the possibility of self-sustaining futuristic smart devices.
6. Several 2D materials can be implanted into Programmable Interface Controllers (PICs), resulting in highly integrated, multifunctional optical devices thanks to recent advances in 2D material production.

## Data Availability

Not applicable.
